# Evolutionary Drivers of Conspicuous Spots in Velvet Ants (Hymenoptera: *Dasymutilla*)

**DOI:** 10.1002/ece3.70896

**Published:** 2025-01-30

**Authors:** Vinicius Marques Lopez, William L. Allen, Mariáh Polido, Lucas Henrique Almeida, Kevin Andrew Williams, Rhainer Guillermo Ferreira

**Affiliations:** ^1^ Graduate Program in Entomology, Department of Biology University of São Paulo (USP) Ribeirão Preto Brazil; ^2^ Lestes Lab Federal University of Triangulo Mineiro (UFTM) Uberaba Minas Gerais Brazil; ^3^ Department of Biosciences Swansea University Swansea UK; ^4^ Centre of Biological and Health Sciences Federal University of São Carlos (UFSCar) São Carlos Brazil; ^5^ Laboratory of Aquatic Biology São Paulo State University (UNESP) São Paulo Brazil; ^6^ Plant Pest Diagnostics Center California Department of Food & Agriculture Sacramento California USA

**Keywords:** animal coloration, antipredator signaling, closed habitats, habitat complexity

## Abstract

Predation plays an important role in animal evolution by selecting for antipredator adaptations. Antipredator color adaptations include conspicuous spots, which are believed to provide protection by deflecting attacks to harmful or peripheral body parts, deimatic signaling, or as conspicuous warning coloration. The utility of antipredator signals is context‐dependent and may be influenced by the environment. In this study, we investigated the selective forces acting on the evolution of conspicuous spots on velvet ants (Mutillidae: *Dasymutilla*). We tested whether conspicuous spots in 80 species of velvet ants evolved in (i) forest‐dwelling species, (ii) habitat‐generalist species, or (iii) species predated by diverse birds and frogs. Results show that conspicuous spots are more likely to evolve in forest‐dwelling species and in areas with more canopy cover, whereas species inhabiting open areas and deserts tend to lose them. Moreover, taxa with conspicuous spots transition between open and forested habitats less often. Spot presence was not associated with predator diversity. We suggest that spots in velvet ants require complex visual environments to be effective, which may limit their habitat occurrence. In simpler environments, carrying conspicuous spots could be costly due to increased exposure to visual predators.

## Graphical abstract

This study investigates the evolution of conspicuous spots in velvet ants (Mutillidae: *Dasymutilla*). We found that conspicuous spots are more likely to evolve in forest‐dwelling species and areas with greater canopy cover, while they tend to be lost in open or desert habitats. We suggest that spots in velvet ants require complex environments to be effective, which may limit their habitat occurrence.

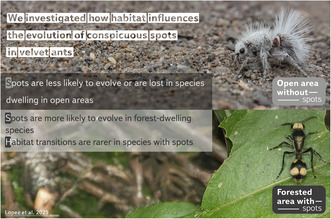

## 1 Background

Predation is a powerful driver of animal evolution; hence, prey possess antipredator adaptations to increase the chances of survival. These adaptations include colors and patterns that implement different antipredator strategies such as camouflage, warning coloration, and deflection (Stevens et al. 2007; Ruxton et al. 2019; Caro and Koneru 2021).

Divergence and convergence of antipredator color phenotypes may arise, driven by the composition of predator communities, their sensory biases, and environmental variables, including the intensity and composition of ambient light, habitat complexity, and structure, as well as prey behavior. Hence, habitat features (at micro‐ and macrohabitat levels) experienced by predators and prey alike can drive the color patterns displayed by prey species (Endler 1992).

Conspicuous spots are isolated distinct circular, oval, or irregularly shaped markings that stand out due to their contrast in color or brightness from the main body, with their effectiveness relying more on their visual conspicuousness than on their specific shape (Stevens and Ruxton 2014). They include and are often referred to as “eyespots” because they are often found in pairs, and their appearance can resemble eyes to a greater or lesser extent (Stevens, Hardman, and Stubbins 2008). However, we reserve the term “eyespots” for cases that display characteristics resembling potential false irises and/or pupils (e.g., Hemingson et al. 2021) and here investigate the larger class of conspicuous spots. Conspicuous spots are observed in many invertebrate and vertebrate taxa, including arthropods, mollusks, amphibians, felids, birds, and fish (Allen et al. 2011; Kane et al. 2019; Crees, DeVries, and Penz 2021; Hemingson et al. 2021; van den Berg et al. 2024).

Conspicuous spots can have a sexual role (Huq, Bhardwaj, and Monteiro 2019; Crees, DeVries, and Penz 2021), but many studies suggest that these patterns can function as antipredator signals via several different mechanisms. For instance, conspicuous spots can act by discouraging and/or intimidating pursuit (Caro 1995; Stevens et al. 2007, 2009; Kjernsmo and Merilaita 2017) or deflecting attacks to either nonvital peripheral regions of the body or those that possess a defense (Stevens 2005; Kodandaramaiah 2011; Vallin et al. 2011; Stevens and Ruxton 2014; Prudic et al. 2015; Ruxton et al. 2019). They may also work as warning colors, signaling predators that prey is defended (Arenas, Walter, and Stevens 2015; Skelhorn et al. 2016). All three mechanisms may involve eye‐mimicry, a compelling hypothesis that suggests that predators perceive conspicuous spots as the eyes of potential enemies (Blest 1957), but for which there is relatively limited evidence (Mizuno et al. 2024; Skelhorn et al. 2016; Skelhorn and Rowland 2022; Stevens, Castor‐Perry, and Price 2009; Stevens and Ruxton 2014). Alternatively, the conspicuous signal hypothesis suggests that conspicuous spots are effective antipredator markings due to their high visibility, making them easy for predators to detect and learn (Stevens and Ruxton 2014). Conspicuousness can promote avoidance, deflect attacks, or reinforce aposematic signals without requiring eye‐mimicry (Stevens, Hardman, and Stubbins 2008; Stevens et al. 2009). Although a great deal of research has investigated spot function and antipredator mechanism, few studies have addressed the selective forces acting on the evolution of conspicuous spots.

One hypothesis is that the efficacy of conspicuous spots varies with the openness of the environment (Hemingson et al. 2021), being more effective in closed environments as forests because these generally contain more diverse background appearances, more diverse light environments (Endler 1992), and more diverse predators (Roll et al. 2017). Conspicuous antipredator traits are thought to be more stable and effective signals to a diverse array of predators against a variety of backgrounds and in variable light environments than camouflage traits where effectiveness is more susceptible to variability in the visual environment and predator community (Merilaita, Scott‐Samuel, and Cuthill 2017; Ruxton et al. 2019; Xiao and Cuthill 2016). Thus, open habitats may favor less conspicuous traits due to a simpler visual environment and generally higher predation pressure, making camouflage a more effective antipredator strategy (see Merilaita and Tullberg 2005; Wheatley et al. 2020; Chang and Todd 2023; van den Berg et al. 2024).

Another hypothesis suggests that during the movement between different habitat types (i.e., displaying habitat generalism), a prey species may face distinct predator communities and environmental contexts. Such behavior could result in convergence on antipredator signals that are common and so recognizable by predators across different communities (Chouteau and Angers 2011). This view is supported by the wide occurrence of conspicuous spots in extant arthropods (Janzen, Hallwachs, and Burns 2010) and paleontological data indicating convergent evolution of conspicuous spots in several fossil taxa (e.g., lacewings and butterflies, see Labandeira et al. 2016). This demonstrates that spots are observed across species with different habits and lifestyles, making it plausible that spots would also be linked to individual generalist species found in diverse habitats.

*Dasymutilla* Ashmead (Hymenoptera: Mutillidae) velvet ants are wasps that occur in many habitats all over Central and North America (see also Bartholomay, Williams, Cambra, et al. 2019) and are brood parasitoids of other insects, mainly wasps and bees. Female wasps are flightless, have a hard exoskeleton, excrete unpleasant smelling chemicals, possess a potent sting, and advertise these defenses using conspicuous aposematic coloration. Velvet ants also use acoustic signals, such as stridulation of their metasoma (i.e., the abdominal region in other insects), as a defense mechanism against vertebrate predators (e.g., Gall et al. 2018; Masters 1980). Female velvet ants form one of the largest sets of Mullerian mimicry complexes in the world, with at least eight mimicry rings described (Wilson et al. 2012), each containing numerous species. Additionally, some females display ultrablack coloration (i.e., colors with extremely low reflectance) adjacent to light patterns, which may increase internal contrast (Lopez et al. 2024). Males can fly but lack a sting and possess a less hard exoskeleton compared to females. However, *Dasymutilla* males often retain some components of the aposematic coloration seen in females, though conspicuous spots are rarely present in any male velvet ant (e.g., Williams 2023). Finally, velvet ant coloration seems to have no function in the mating behavior (VanderSal‐Jensen, Crews, and Gillespie 2016).

**FIGURE 1:**
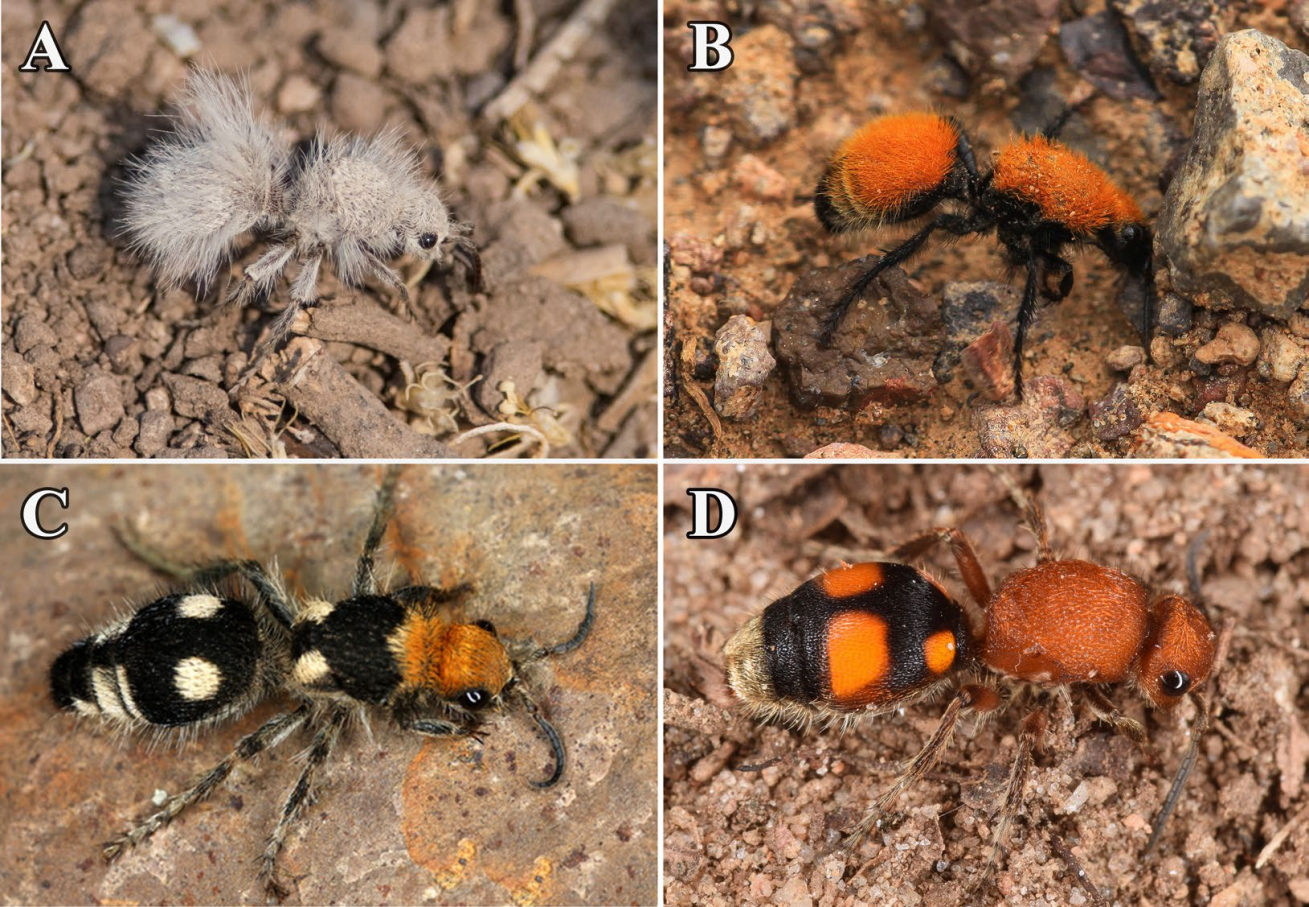
Color patterns of velvet ants in nature against typical natural backgrounds. Pictures show examples of species without conspicuous spots (A and B) and species that have conspicuous spots (C and D). (A) *Dasymutilla gloriosa*, (B) *D.* *bioculata* , (C) *D. araneoides*, and (D) *D. quadrigutatta*.

Conspicuous spots of velvet ants are typically concentrated on the metasoma, which may deflect predator attacks to a less critical region for survival (Kodandaramaiah 2011; Stevens 2005) (Figure 1). Additionally, the metasoma of velvet ants is where an array of defenses is concentrated (i.e., painful sting, hard cuticle) (Deyrup 1988), thus potentially drawing predators toward dangerous defenses or simply helping to advertise their presence to facilitate learning by predators. Velvet ant conspicuous spots may also simply act as a warning signal. This suggests that conspicuous spots in velvet ants, although lacking features typically associated with eye‐mimicry, may function as salient antipredator signals. Here, we used comparative analyses controlling phylogenetic relationships to assess the association between conspicuous spots, habitat, and predator community in velvet ants. Specifically, we tested two separate hypotheses: (i) whether conspicuous spots evolved in forest‐dwelling species and (ii) whether they evolved in habitat‐generalist species. Additionally, we investigated the role of the light environment and predator community by examining the relationship between conspicuous spots, canopy cover, and the diversity of anurans and avians.

## 2 Results

In total, 80 species of velvet ants (i.e., *Dasymutilla*) were analyzed, representing 52% of described species. The classification of spots resulted in a substantial level of interobserver agreement (Kappa coefficient = 0.790). We identified 39 species with spots (48.7%). Among the habitat classifications, 36 species (45%) were identified as forest‐dwellers, and 62 species (77.5%) occurred in open areas, with some species inhabiting both environments. In total, 32 (40%) were habitat generalists, and 48 (60%) were specialists in only one habitat type. Furthermore, the diversity of predator species (i.e., maximum and minimum values) varied from *Traumatomutilla* sp. (outgroup and forest dwelling) (Avian = 249.8 species, Anurans = 53.7 species) to *D. militaris* (forest dwelling) (Avian = 57.6 species) and *D. scabra* (open dwelling) (Anurans = 2.5 species). The percentage of canopy cover for each species ranged from 40.3% (*D. chalcocephala*) to 0.01% (*D. nocturna*). On average, forested habitats showed a higher predator diversity compared to open habitats. Specifically, avian diversity in forested areas was higher (Avian = 113.17 species ±35.82) than in open areas (Avian = 99.39 species ±20.23), and anuran diversity was also greater in forested habitats (Anurans = 15.90 species ±8.87) compared to open areas (Anurans = 11.52 species ±5.39). Canopy cover in forested areas was significantly higher (17.77% ± 11.75%) than in open habitats (9.10% ± 9.10%).

**FIGURE 2:**
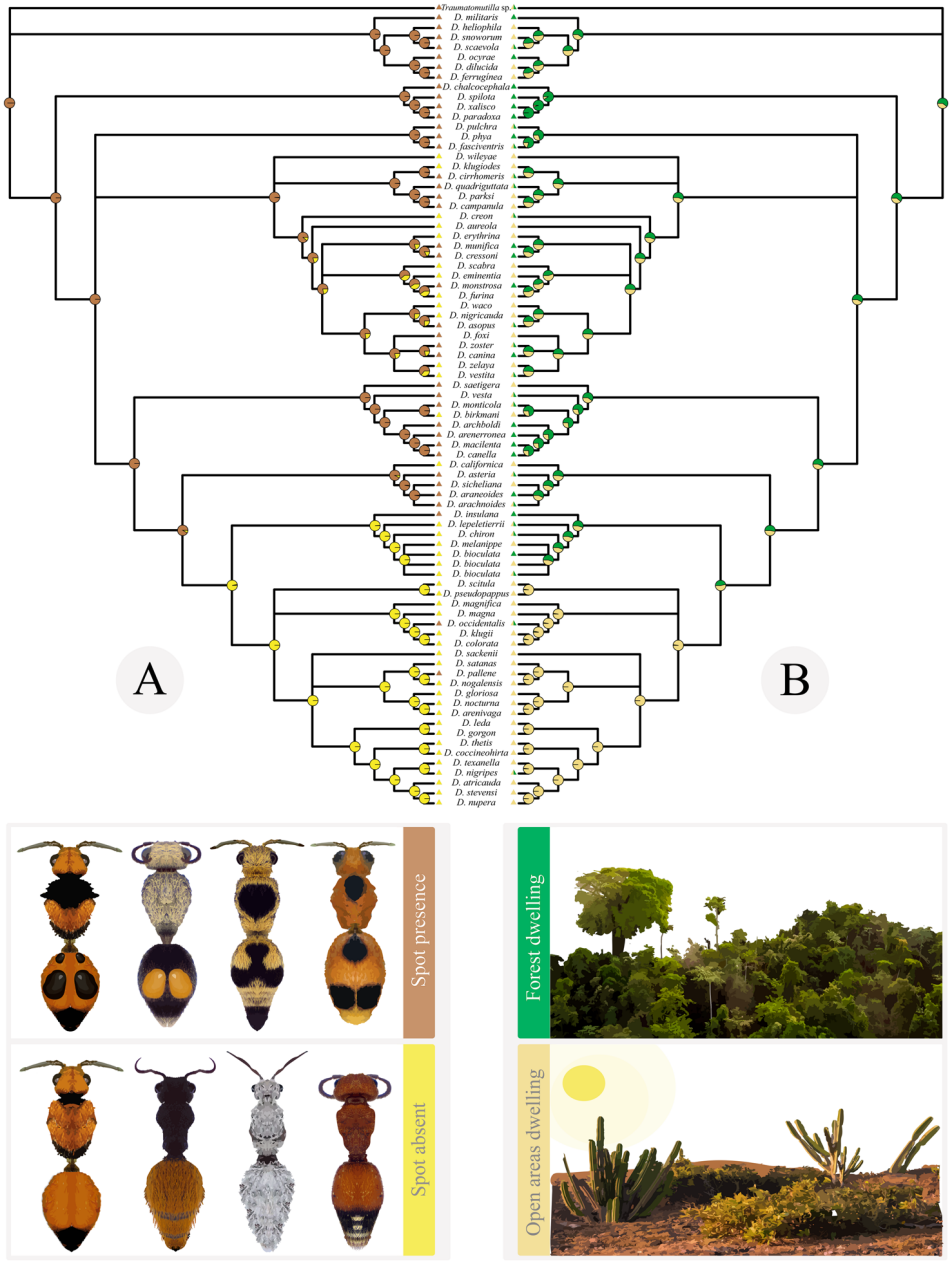
Ancestral state reconstruction for spots (A) and forest‐dwelling (B) in *Dasymutilla*. (A) Presence and absence of spots are represented in brown and yellow, respectively. (B) Occurrence in forests is represented in green, and absence in forested areas is represented in beige.

The analysis of phylogenetic signal revealed that there is a strong phylogenetic structure associated with the presence of conspicuous spots (*D* = 0.1). This deviates significantly from the expectation of a random phylogenetic pattern that is consistent with the expectation under a Brownian model of evolution (Fritz and Purvis 2010). Ancestral state reconstruction suggested spot presence, forest habitat (Appendix S1: Table S1; Figure 2), and habitat specialists were the ancestral conditions in *Dasymutilla* (Appendix S1: Figure S1 and Table S1).

**FIGURE 3:**
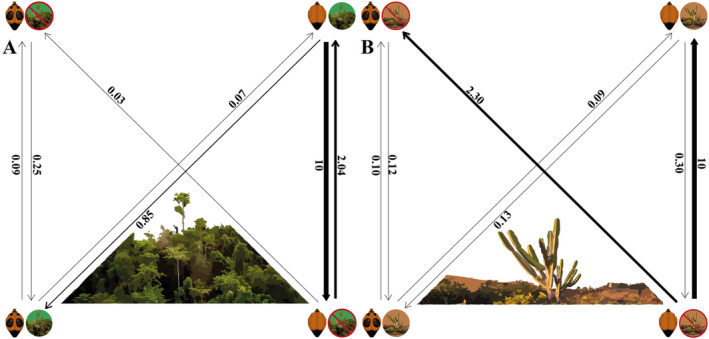
Evolutionary transition rates of spots and habitat characteristics in velvet ants. (A) Conspicuous spots and forested areas; (B) spots and open areas. (A) Green circles denote presence in forests, and green circles outlined in red indicate absence in forests. (B) Brown circles signify presence in open areas, and circles with red outlines indicate absence in open areas. The presence or absence of conspicuous spots is illustrated on the metasoma of each velvet ant depicted. Arrows indicating transition rates of < 0.5 per million years are shown as thin lines, transition rates between 0.5 and 2 as regular lines, transition rates between 2 and 3 as bold lines, and transition rates > 3 as the thickest lines.

On testing for correlated evolution of spots and habitat characteristics (open or forested), there was strong support for the correlated model (AICw = 0.97, Table 1). Analysis of transition rates showed this was due to a positive correlation between conspicuous spot presence and forest‐dwelling (Figure 3A). In support of our prediction, the acquisition of conspicuous spots is more frequent in forested habitats compared to open habitats (0.85 vs. 0.03 trans/MY), and transitions to forest habitats are more frequent when a lineage possesses spots (0.25 vs. 0.09) (Figure 3A). Lineages transition away from forest habitats near‐instantaneously when they lack spots, faster than the (high) rate at which they transition to forest habitats (10 vs. 2.04) (Figure 3A). In summary, overall, the evolutionary association between spots and forest habitats is strong (Tables 1 and 2).

**TABLE 1:** Akaike Information Criterion (AIC) scores for evolutionary models between spots and habitat characteristics (open or forest environment) and habitat occupancy (generalist or specialist species). Bold indicates the model with the lowest AIC. AICw, Akaike weight.

Markov model	Forest/open		Generalist/specialist	
	AIC	AICw	AIC	AICw
Independent	219.3	0.00	**206.6**	0.85
Correlated	**197.8**	0.97	210.3	0.13
Hidden Markov independent	205.0	0.02	215.5	0.01
Correlated hidden Markov	209.8	0.00	221.9	0.00

**TABLE 2:** Phylogenetic logistic regression results for evolutionary model between spots and light conditions (tree cover) and predator diversity (anuran and avian). Significant results in bold.

Variables	Estimate	SE	*t*	*p*
Tree cover	0.02	0.04	5.93	**< 0.0001**
Anuran diversity	0.04	0.07	0.61	0.54
Avian diversity	−0.01	0.10	−0.14	0.88

The correlated model also showed the most robust support for open areas (significant weight, AICw = 0.88) (Appendix S1: Table S2). Lineages without conspicuous spots exhibited a significantly higher rate of acquiring the transitioning to open areas (10 vs. 0.30 trans/MY) (Figure 3B) compared to lineages with conspicuous spots gaining the same trait. This implies that spots might be less advantageous or even disadvantageous in open areas, potentially leading to their lower persistence in such environments (Figure 3B). Interestingly, species without conspicuous spots seem to exhibit generally faster transition rates overall (Figure 3A,B). Additionally, it is noteworthy that the loss of spots happens at a considerably slower rate (0 and 0.09) compared to gaining spots (2.3 and 0.13) (Figure 3B). There was no evolutionary association between spot presence and habitat generalism or specialism; the independent evolutionary model was best supported (Table 1). We have included the evolutionary transition rates for spots and species categorized as generalists/specialists in the (Appendix S1: Figure S2).

Our analysis of canopy cover using phylogenetically controlled logistic regression models indicates that spots were significantly associated to areas with a higher tree cover (i.e., more forested environments) (Table 2), supporting the association between spots and forest habitats (Table 1). In contrast, predator diversity was relatively unimportant in predicting the occurrence of spots in velvet ants (Table 2).

## 3 Discussion

Here, we have shown that the presence or absence of spots in velvet ants is associated with their habitat characteristics. Our findings suggest that the spots tend to be lost or do not evolve in species that dwell in open areas and deserts, whereas they are more likely to evolve in forest‐dwelling species. This compellingly indicates that the features of the habitat may drive the evolution of this antipredator trait. Results suggest that velvet ants without spots exhibit more frequent transitions between open and forest environments, while their spotted counterparts seem to have a lower lability in terms of habitat characteristics. We also show that habitat generalism and predator diversity do not reliably predict the presence or absence of spots. These results suggest that the species' habitat occupancy does not drive the evolution of spots.

### 3.1 Why Do Forest Dwellers Have Spots?

Our results support the hypothesis that forests may favor the evolution of spots as antipredator color traits. Velvet ants, known for their agility and mobility, are cursorial species that traverse various microhabitats. Thus, those that inhabit forest habitats face a plethora of selective pressures because of the diverse microenvironments which they utilize and cross through, which differ substantially from open expanses and deserts. Forested domains are characterized by a heightened level of both structural intricacy and heterogeneity, in terms of physical structure, visual background, and light environment (Endler 1993). Complex habitats (e.g., forests) also can provide numerous hiding places for prey while creating physical barriers that hinder predator movement, thereby making it more difficult for predators to locate and capture their prey (Gaigher, Pryke, and Samways 2024). Thus, it seems reasonable to expect that, in forest regions, natural selection would reward the adoption of a conspicuous trait that serves as an effective aposematic signal to a diverse array of predators, in other words, a signal that retains its effectiveness as a conspicuous and memorable signal against a variety of backgrounds.

Recent evidence supports this hypothesis (Hemingson et al. 2021). Planktivorous pelagic fish species tend not to have eyespots, while their reef‐dwelling counterparts carry eyespots. This possibly occurs due to heightened exposure to visual predators in pelagic fish that inhabit an open environment, when compared to the reef fish that can hide in a more heterogeneous habitat (Hemingson et al. 2021). This finding suggests that spots may be more efficacious in species that are harder to locate owing to their habitat selection behaviors favoring complex habitats (Hemingson et al. 2021). Thus, the loss of spots in velvet ants may be a direct outcome of evolutionary transitions to open and desert habitats. In such circumstances, conspicuous spots in velvet ants may have been lost to adapt to increased exposure to visual predators. However, differences between velvet ants and reef fish also need to be considered. Unlike velvet ants, most reef fish do not possess a significant secondary defense (such as a painful sting). In cryptobenthic reef fish, the eyespot is typically positioned near the head, implying a function in intimidation or mimicry (Hemingson et al. 2021). Conversely, in more active species, eyespots are commonly found toward the rear of the dorsal fin, indicating a role in deflection (Hemingson et al. 2021). Therefore, any comparisons made between these two organisms must be approached with caution and taken in the context of their unique ecological and evolutionary histories.

### 3.2 Habitat Generalism Is Not Associated With Spots

Drawing upon the same principle of predator and background diversity, it follows that organisms utilizing varied habitats might be more likely to acquire a conspicuous trait that reduces the probability of predation in a variety of habitats. However, our findings do not support the notion of habitat generalism favoring the evolution of spots. Current evidence is mixed with regard to whether specialists or generalists tend to have more conspicuous defenses. For instance, in ladybirds, species that specialize in particular habitats exhibit a greater degree of conspicuousness than their generalist counterparts (Arenas and Stevens 2017). On the other hand, evidence suggests that generalist species are less likely to adopt camouflage than specialists as they experience a more diverse range of background appearances, which a camouflage strategy struggles with (Elias, Anker, and Gawryszewski 2019; Endler 1984; Hughes, Liggins, and Stevens 2019; Speed, Brockhurst, and Ruxton 2010). Therefore, the relationship between habitat generalism/specialism and the evolution of spots and other antipredator color traits is inconclusive, and our results cannot help resolve this question.

### 3.3 Predator Diversity Is Not Associated With Spots

Conspicuous colors in female velvet ants can play an important role in predator education, fostering negative associations with their potent secondary defenses, such as the intense pain caused by their sting (Gall et al. 2018). This process can accelerate the speed and accuracy of decision‐making in predators when they encounter similar color patterns (Gall et al. 2018; Mergler and Gall 2021). However, observational and experimental evidence on predation of velvet ants is scarce, leaving the identity of their natural predators largely unknown (Schmidt, and Blum 1977; Gall et al. 2018; Mergler and Gall 2021). In a controlled experiment that exposed *Dasymutilla* to various insectivorous vertebrates, only a single American toad ( *Anaxyrus americanus* , Bufonidae) was able to capture a velvet ant but at the cost of sustaining injury during the encounter (Gall et al. 2018). Other toads, as well as lizards, shrews, birds, and a mole all failed to successfully attack velvet ants. Predatory birds, including those known to feed on defended insects like bees and wasps, exhibited strong aversions, avoiding interactions not only with live *Dasymutilla* females but also with dead specimens and mealworms painted to mimic the conspicuous colors of velvet ants (Gall et al. 2018). Thus, the rarity of documented predation events in the lab and wild complicates our understanding of how these signals influence predator behavior in natural environments (Akcali et al. 2019; Smith et al. 2020).

Although we did not investigate mammal or reptile diversity, our results suggest that predator diversity does not correlate with the presence of conspicuous spots in velvet ants. The concept of a “defense portfolio” (Kikuchi et al. 2023) offers a plausible explanation for why conspicuous spots alone may not be associated with predator diversity. As Kikuchi et al. (2023) emphasize, prey often deploy multiple defenses, either simultaneously or sequentially, across different stages of the predation sequence, such as detection, identification, attack, and consumption (see also Caro 2005). This defense portfolio allows prey to engage various mechanisms to survive predation, rather than relying on a single defensive trait (Kikuchi et al. 2023). In velvet ants, conspicuous coloration may serve as an initial warning (a primary defense), while other defenses, such as potent sting or mechanical resistance, act in later stages (secondary defenses), ensuring multiple layers of protection. This synergy between antipredator traits implies that conspicuous spots alone may not predict predation outcomes, as they function within a broader suite of defenses that collectively deter a wide range of predators. Rather than implying a direct adaptation to specific predator types, conspicuous spots in velvet ants may act as a warning signal within this multimodal defense system, complicating the relationship between signal expression and predator diversity (see review in Kikuchi et al. 2023). Consequently, our results can be interpreted as reflective of selection pressures tied to predation, acknowledging the complexity of predator–prey interactions and suggesting that conspicuous spots may still provide a selective advantage despite the absence of a clear correlation with predator diversity.

### 3.4 Is it Possible for Conspicuous Spots to Aid in Camouflage?

Conspicuous patterns, characterized by vibrant colors and high‐contrast designs, are believed to increase signal salience against diverse backgrounds (e.g., Stevens and Ruxton 2012). However, the effectiveness and function of these patterns may vary based on a complex interaction between the animal's coloration, environmental features of the visual background, and visual and cognitive capabilities of different predators. Consequently, conspicuous patterns may serve not only to warn predators but also to obscure prey, depending on the context.

The function of defensive coloration may be distance‐dependent (Barnett and Cuthill 2014). In the cinnabar moth caterpillar, although the orange and black stripes are highly visible up close, they blend seamlessly with the surrounding environment when viewed from a distance (Barnett, Cuthill, and Scott‐Samuel 2018). Similarly, spotted skunks can appear cryptic under certain lighting conditions when seen from afar but become highly visible up close (Caro et al. 2013). In certain microhabitats, high‐contrast patterns may function as disruptive camouflage (Honma, Mappes, and Valkonen 2015). Additionally, factors such as prey posture and the predator's viewing angle can influence detectability (Cezário et al. 2021; McEwen et al. 2024). Thus, conspicuous patterns can provide dual functions depending on the context, offering an additional layer of defense against visually adept predators and enhancing the overall effectiveness of the signal (e.g., Barnett et al. 2017). In velvet ants, camouflage has been proposed to explain body color lightness across environments with varying tree cover (Lopez et al. 2021). Therefore, it is possible that conspicuous spots may contribute to camouflage under certain conditions. Further studies are needed to assess this potential function.

### 3.5 Spots and Mimicry Rings

Conspicuous spots may serve not only as direct antipredator strategies but also as key components of mimicry rings, where multiple species—often coexisting in the same environments—develop similar warning signals to gain collective protection from possible predators (e.g., Chan et al. 2021). For instance, mimicry rings featuring conspicuous spots are found in velvet ants across the globe (e.g., Wilson et al. 2012, 2015, 2018; Cambra, Brothers, and Quintero 2016; Lopez et al. 2018, 2019; Bartholomay, Williams, Lopez, et al. 2019; Waldren et al. 2020; Lelej et al. 2023; Okayasu 2023; Boutin and Vilhelmsen 2024). Thus, the way predators use the habitat affects the selective pressures on mimetic prey. This process favors the convergence of antipredator color traits and strategies (Chouteau and Angers 2011; Gompert, Willmott, and Elias 2011; Willmott et al. 2017). Additionally, in species‐rich communities where prey types are relatively evenly distributed, predators are more likely to generalize avoidance across broadly similar prey types (Beatty, Beirinckx, and Sherratt 2004; Kikuchi et al. 2019). This increases the chances that predators will rely on a “key” characteristic to group prey into broad categories (Kikuchi et al. 2019, 2021).

Conspicuous spots are a widespread antipredatory strategy observed across a broad range of taxa, including assassin bugs, beetles, butterflies, mantids, moths, spider wasps, spiders, and velvet ants (Bartholomay, Williams, Cambra, et al. 2019; Chan et al. 2021; Evans 1950; Mawdsley 1994; Nentwig 1985; Stevens 2005; Zhang and Weirauch 2011). This suggests a convergent evolutionary strategy, where carrying the appropriate conspicuous signal offers an adaptive benefit by either warning, startling, or deterring predators through innate or learned responses (Janzen, Hallwachs, and Burns 2010). In some cases, conspicuous spots—such as eyespots—can also deflect or intimidate predator attacks (Stevens 2005). In female velvet ants, the presence of conspicuous spots, combined with other defensive traits such as a sting, hard cuticle, and metasomal stridulation, may reinforce the role of an antipredator signal. This combination not only contributes to individual defense through a multicomponent, multimodal display but also reinforces the signal's impact across a broader prey community due to its frequency. Similarly, the efficiency of a warning signal increases proportionally with its prevalence within the natural community reaching a threshold where protection stabilizes (Chouteau, Arias, and Joron 2016). This phenomenon aligns with positive frequency‐dependent selection, indicating that predator knowledge becomes saturated only for the most common warning signals, increasing the signal's effectiveness (Chouteau, Arias, and Joron 2016). In other words, a female velvet ant with spots can use an effective antipredator strategy as it moves between forests because the same strategy is present in the wider local mimetic prey community.

### 3.6 A Combination of Environment and Mimetic Rings?

Habitat characteristics seem to drive the evolution of antipredator traits in velvet ants, with conspicuous spots more likely to evolve in forest‐dwelling species. Species in the same scenario may converge to adopt similar color patterns according to the cost effectiveness of each habitat–strategy relationship, forming mimicry rings. For example, velvet ants in open areas may exhibit lighter colors and reddish tones (Lopez et al. 2021) which may aid in camouflage (Endler 1993). In forested habitats, darker integument hues are offset by striking spots in shades of red, orange, white, and yellow. Hence, the selection pressures that are driving the evolution of velvet ant spots may be disruptive in nature. These pressures might accentuate certain conspicuous spots in darker and more intricate environments while promoting the loss of these traits in clearer and simpler environments. Membership of a mimicry ring would reinforce this trend, driving antipredator colors toward convergence in each habitat type (open/forest). Our results suggest that the adoption of spots in such mimicry rings and forests may limit the evolutionary lability of these taxa in terms of transitioning between forest and open habitats.

Environments can impact the detection and effectiveness of aposematic signals in different ways. For instance, in environments with greater phenotypic diversity among prey, like forests, there is a higher chance that predators will use a “key” characteristic to identify unprofitable prey (Kikuchi et al. 2019). This, in turn, may favor the evolution of mimicry rings (Kikuchi et al. 2019). Additionally, changes in light conditions may also affect the predator behavior and foraging decisions (Nokelainen et al. 2022). Studies in nature, using wax models with different color patterns, that is, aposematic versus cryptic colors, showed that the detectability of different morphs depends on both predator experience and luminosity (Rojas, Rautiala, and Mappes 2014). Moreover, colors like red, orange, and yellow—which are the primary colors of spots in velvet ants' mimetic rings—offer high contrast and maintain their stability even under diverse environmental conditions and throughout the day (Arenas, Troscianko, and Stevens 2014). Therefore, natural selection can lead to the evolution of more effective aposematic signals in certain environments shaped by the predator communities typical of these habitats. As a result, mimicry rings may develop, and species may experience reduced lability to changes in habitat characteristics (e.g., reducing transition rates to open habitats).

## 4 Conclusions

In sum, species that inhabit forests tend to evolve spots, while their open‐area and desert‐dwelling counterparts tend to lose spots. The emergence of conspicuous spots among velvet ants may be attributed to selective forces that arise from habitat characteristics, including background appearance, predator communities, and local mimetic rings. Spots may also limit evolutionary transitions in habitats with different characteristics, while species without conspicuous spots show an evolutionary history with higher lability in forest/open area habitat. Nevertheless, it is important to underscore that our correlative analyses do not infer any causal relationship. Furthermore, we recognize that we have not yet identified the primary driver behind the evolution of conspicuous spots in velvet ants, and it is likely that additional mechanisms are involved. New research is needed to explore these dynamics, particularly through experiments that evaluate how and to what extent factors such as ambient light, resource availability, predator types, habitat complexity, and other characteristics of forest environments influence the presence of conspicuous spots in velvet ants.

In forests, spots may startle the predator, deflect attacks to defended body regions in females, or form an aposematic signal associated with defenses. These defenses may be especially effective in forest environments because the prey may find it easier to escape in the complex scenario of the background (leaves, twigs, etc.) hampering repeat attacks. On the other hand, desert and open area species are exposed in a more visually assessable environment, where predators have a chance to get closer, inspect their prey, and attempt multiple attacks, all while having ample time to form a searching image. This makes having conspicuous spots a costly strategy in open areas, so camouflage would be a more advantageous strategy. In open areas, long setae may have substituted the spots as predator deterrence strategy at close range while maintaining camouflage (e.g., Wilson et al. 2020). Further research is essential to understand how environmental complexity and prey mobility influence survival under predation pressure.

We posit that the protective colors that accompany the manifold predator deterrence strategies of velvet ants qualify them as exemplary models for filling gaps in the field of animal coloration. Given that these visually striking creatures also constitute one of the largest mimicry rings in the natural world (Wilson et al. 2012), future inquiries ought to concentrate on unraveling the co‐occurrence of these strategies among Müllerian and Batesian mimics in forests and open areas.

## 5 Material and Methods

### 5.1 Image Data and Spot Measurement

The color patterns of velvet ants were evaluated using photographs of adult female specimens in entomological collections. All photos were of specimens in museum collections taken by one of us (KAW). To classify conspicuous spot presence on velvet ants, we considered only markings present on the metasoma (i.e., the abdominal region in other insects) (Figure 1). We defined conspicuous spots as circular marks, united circular marks, or diagonal marks that occur on the first and/or second metasomal segments and contrasted in color or lightness from the overall metasoma color. We focused on the metasoma only because that is where female defenses are concentrated.

Naïve (MP) and non‐naïve (VML, a specialist on velvet ants) evaluators classified the color patterns separately, utilizing dorsal photographs to quantify the conspicuous spot marks of each species. When there was disagreement among the observations, we chose to use the naïve observer classification. We used kappa coefficient to quantify the agreement between the classification of the two independent observers (Landis and Koch 1977).

### 5.2 Ecological Data

To study the relationship between conspicuous spots and habitats, we classified the ecological information for each species into two predictor variables: (i) forest or open environment and (ii) habitat‐generalist or specialist. We considered open and forest environments as proxy of habitat characteristics, and generalist or specialist as indicative of habitat occupancy. First, we collected information on habitat(s) in which the species occur using specialist knowledge, species descriptions, and available literature, supported by information from collections, field observations, and biodiversity websites (i.e., inaturalist.org). We utilized iNaturalist due to its status as the most comprehensive and representative database for *Dasymutilla*. Moreover, all identifications are verified by experts, enhancing the reliability of the data.

We considered a set of nine habitats that embrace the whole distribution of our studied species across North and Central Americas: (i) Humid Tropical Forests; (ii) Dry Tropical Forests; (iii) Northwestern Forested Mountains; (iv) Eastern Temperate Forests; (v) Caribbean region; (vi) Southern Sierras; (vii) Mediterranean California; (viii) Great Plains; and (ix) North American Deserts. A value was assigned for each species considering its absence (0) or presence (1) in each habitat type (see table in Appendix S2). We determined species as utilizing closed habitats if there was at least one occurrence in forests (i.e., from “i” to “v”), while species classified as utilizing open habitats had at least one occurrence in open fields and deserts (from “vi” to “ix”). A species could be scored as both depending on its distribution and habitat preferences. As only four species were classified as inhabiting more than two habitat types, we classified habitat generalists as species that occur in more than one habitat (computed with a score of 1), while species that occurred in only one habitat were classified as specialists (scored as 0). KAW classified the ecological data for all species.

While expanding our variables to include multiple categories (e.g., open/semi‐open/forest) might potentially improve the classification accuracy, we chose to maintain them as binary characteristics for different reasons. Concerning the forest/open variable, this choice was prompted by the ambiguity and subjectivity inherent in the term “semi‐open,” which exhibits considerable variation depending on the context and individual interpretation. Furthermore, the complex transition between open and forested habitats in various ecosystems presents difficulties in precisely delineating boundaries. In the case of the generalist/specialist variable, our dataset predominantly consists of species found in one or two habitats, with only a small number of species occurring in more than two habitat types (< 4 species). Therefore, to address concerns related to subjectivity and statistical robustness, we opted to utilize binary variables.

Our second dataset comprised data on canopy cover, anuran diversity, and avian diversity obtained from a spatial analysis of species' ranges in relation to predictors using the Inaturalist.com database and personal data.

We utilized tree cover layers (Sexton et al. 2013) as a representation of light environment. Additionally, we chose the diversity layers of Passeriformes and Anura (Jenkins, Pimm, and Joppa 2013; Pimm et al. 2014, available at: https://biodiversitymapping.org/) as proxies for predator diversity. Predation records for velvet ants in the field are scarce (Schmidt, Schmidt, and Schmidt 2021). In controlled experiments, velvet ants were exposed to various potential predators, including frogs, lizards, birds, and small mammals (Gall et al. 2018). In all interactions, the velvet ants survived, and predators learned to avoid them in subsequent attempts (Gall et al. 2018). The literature identifies frogs (Anura), lizards, and birds (Passeriformes) as the primary potential predators of velvet ants (Vitt and Cooper 1988; Manley and Sherbrooke 2001; Gall et al. 2018; Sugiura 2020; Mergler and Gall 2021; Schmidt, Schmidt, and Schmidt 2021). Due to the uncertainty surrounding the specific predators of Mutillidae, we focused on two taxa with the available global diversity data: avians and anurans. We selected these groups because, at present, no global diversity layers for other potential predator groups, such as lizards, are available. All points and identifications were verified by one of us (KAW). After data extraction, we excluded species with fewer than five coordinates (*N* = 5) (see Appendix S1: Table S3).

For the extraction of predictor variables, we followed the methodology outlined by Lopez et al. (2021). Variables were individually extracted within an 11 km diameter buffer around the specimen's occurrence point using the point radius method (Wieczorek, Guo, and Hijmans 2004; Lopez et al. 2021). This buffer size was chosen as it is the most appropriate for addressing uncertainties in the occurrence point (e.g., data derived from citizen science). Studies have shown that buffers around 10 km do not incur distortions in results when analyzing the spatial distribution of species (e.g., Fernandez et al. 2009). In cases where the location occurred in an urbanized environment, we standardize the position of the point in the non‐urbanized area closest to the centroid of the location (i.e., original location), considering the natural conditions of host occurrences (Wieczorek, Guo, and Hijmans 2004; Lopez et al. 2021). All variable extractions were performed utilizing Google Earth Engine (GEE) (Gorelick et al. 2017). The buffer (11 km diameter) was created around the specified layers. We then calculated the mean of each variable for each individual using a zonal statistics approach (Gorelick et al. 2017). The GEE code is provided in the Mendeley repository.

### 5.3 Phylogenetic Tree

*Dasymutilla* comprises a total of 152 valid species, although approximately half of them do not have any molecular information available. Therefore, for our study, we exclusively relied on the subset of 80 species, with sequence data available in GenBank. One species ( *D. bioculata* ) exhibits three distinct morphs, which we considered as separate terminal taxa or “species”.

A similar dataset, consisting of females from 65 Dasymutilla species, was utilized by Wilson et al. (2012) for phylogeny construction. Our dataset includes the 65 species used by Wilson et al. (2012) and an additional set of subsequently deposited sequences adding a further 15 species. We reconstructed a phylogeny using sequence information on Genbank following the methodology and parameters outlined by Wilson et al. (2012). To obtain their phylogeny, we reconstructed a phylogeny using sequence information on Genbank following the methodology and parameters outlined by Wilson et al. (2012). To align with Wilson et al. (2012), we designated *Traumatomutilla* as the outgroup in our phylogenetic analysis, as it has consistently been recovered as the sister group to *Dasymutilla* (e.g., Waldren et al. 2023). Our findings closely align with the topology presented by Wilson et al. (see Figure 3 in the work of Wilson et al. 2012).

To reconstruct the phylogenetic hypothesis, the analysis comprised molecular markers, including two noncoding markers (ITS1 and ITS2) and the wingless gene (see GenBank codes in Appendix S1). The evolutionary models used for wingless gene, ITS1, and ITS2 were SYM + I + Γ, GTR + Γ, and GTR + Γ + I, respectively. To perform Bayesian analysis, we used MrBayes 3.2.2 (Ronquist et al. 2012) with four independent Monte Carlo–Markov chain runs for 5,000,000 generations, with sample frequency of 1.000, discarding the first 10% of generations as burn‐in. Finally, branch support was assessed by posterior probability (PP), presented on a 50% majority consensus tree.

To time‐calibrate the phylogeny, we utilized the “makeChronosCalib” function from the “ape” package (Paradis et al. 2019). This involved specifying the root node and inputting the minimum and maximum ages of the ancestral node based on the oldest available fossil record of *Dasymutilla*, *D. albifasciatus*, with age constraints set between 13.7 and 20.4 million years (Manley and Poinar Jr. 1991, 1999; Waldren et al. 2023). Subsequently, the “chronos” function was employed to perform time calibration (Paradis et al. 2019). All analyses were performed using the R environment (R Core Team 2022).

### 5.4 Correlation and Evolution Between Habitat and Spots

Classic approaches for testing evolutionary correlations between two discrete characters (Pagel 1994) have been reported to have elevated type 1 error rates (Maddison and FitzJohn 2015). Recently, Boyko and Beaulieu (2023) demonstrated that the statistical solution to this problem lies within the expanded model space provided by the Hidden Markov Model (HMM) framework. Thus, we assessed the evolutionary relationship between the presence of conspicuous spots and habitat characteristics (forest or open environment) and habitat occupancy, that is, generalist or specialist, by assessing the evidence for correlated evolution under the new multirate independent model proposed by Boyko and Beaulieu (2023).

We employed the “fitCorrelationTest” function from the “CorHMM” package (Beaulieu et al. 2022), which automatically fits a set of independent and dependent models to test the correlation between our characters. We used the Akaike Information Criterion (AIC) and Akaike weight (AICw) values to rank the best‐fitting model. The transition rates between the character states of the best‐fitting model were then used to interpret character evolution. Finally, the “ancRECON” function in “CorHMM” package was utilized for ancestral state reconstruction analyses for each of the variables: (i) forest dwellers; (ii) generalist/specialist; and (iii) conspicuous spots.

Species found in both forested and open habitats were categorized as “forest” in our study because they experience the habitat heterogeneity hypothesized to promote conspicuous spot evolution. In the main Results section, we present findings based on this classification (see section 5.2). To verify whether this decision impacted results, we re‐ran analyses where these species were designated as “open”. We have included these analyses in Appendix S1. All analyses were conducted using the R environment (R Core Team 2022), and both the data and scripts are available in the Mendeley repository (https://doi.org/10.17632/dt7d7zjc9p.4).

### 5.5 Logistic Regression Analyses

To assess the relationship between conspicuous spots and canopy cover and predator diversity, we conducted phylogenetic logistic regression analyses using the “IG10” method developed by Ives and Garland Jr (2010). This method, implemented through the “phyloglm” R package, incorporates a correlation matrix to consider the phylogenetic relatedness among taxa within the Generalized Linear Model (GLM) framework for binomial distributions (Ho et al. 2022). We used the binary dependent variable representing the presence or absence of conspicuous spots. A single model was constructed incorporating three continuous predictors: Percentage of canopy cover, anuran diversity, and avian diversity.

To standardize the data, we applied *Z*‐Score standardization, which converts the raw values into a common scale (Legendre and Legendre 2012). This process involved centering the data by subtracting the mean of each variable and then scaling by dividing by the standard deviation. The standardized values allow for the comparison of variables that were initially on different scales (Schielzeth 2010; Dalal and Zickar 2012). The transformation was performed using the “scale” function in R. The standardized dataset was then used for subsequent phylogenetic logistic regression analyses, as well as tests for assessing assumptions and diagnosing the logistic regression models.

To ensure the validity of our logistic regression analyses, we performed thorough tests to assess model assumptions and diagnose potential issues (in Kassambara 2018). The linearity assumption was evaluated by comparing the logit of the predicted probabilities against each predictor variable. Smoothed scatter plots indicated an approximate linear association with the predictors, supporting the model's linearity assumption (Appendix S1: Figure S3). Influence analysis revealed no significant influential values; Cook's distance values were all below 1 (Appendix S1: Figure S4A), and standardized residuals remained under 3, indicating no outliers. Additionally, variance inflation factor (VIF) values were assessed to check for multicollinearity among predictors. The following values were observed: tree cover (1.026), anuran diversity (1.044), and avian diversity (1.019). These values suggest no significant multicollinearity issues (Appendix S1: Figure S4B). All data and R codes are provided in the Mendeley repository.

### 5.6 Phylogenetic Signal

To explore the phylogenetic signal associated with the presence of conspicuous spots, we used Purvis' D statistic (Fritz and Purvis 2010), implemented in the phylo.d function from the “caper” package in R (Orme et al. 2013). This method compares the sum of changes in the estimated nodal values of a binary trait (such as the presence or absence of conspicuous spots) along the branches of the phylogeny against what would be expected under a random phylogenetic pattern or a Brownian evolution threshold model. *D* value close to 0 indicates that the trait has evolved under a Brownian motion model, suggesting a few origins, followed by subsequent diversification. In contrast, a *D* value close to 1 would indicate that the evolution of the trait is random with respect to phylogeny, supporting the idea of multiple independent origins.

## Author Contributions

**Vinicius Marques Lopez:** conceptualization (equal), data curation (equal), formal analysis (equal), methodology (equal), visualization (equal), writing – original draft (equal), writing – review and editing (equal). **William L. Allen:** conceptualization (equal), formal analysis (equal), methodology (equal), supervision (equal), writing – original draft (equal), writing – review and editing (equal). **Mariáh Polido:** data curation (equal), validation (equal). **Lucas Henrique Almeida:** formal analysis (equal), validation (equal), visualization (equal), writing – review and editing (equal). **Kevin Andrew Williams:** data curation (equal), formal analysis (equal), funding acquisition (equal), validation (equal), writing – original draft (equal), writing – review and editing (equal). **Rhainer Guillermo Ferreira:** conceptualization (equal), formal analysis (equal), funding acquisition (equal), methodology (equal), supervision (equal), validation (equal), writing – original draft (equal), writing – review and editing (equal).

## Consent

The authors have nothing to report.

## Conflicts of Interest

The authors declare no conflicts of interest.

## Supporting information


Appendix S1.

Appendix S2.

## Data Availability

Lopez et al. (2024), “Evolutionary drivers of conspicuous spots in velvet ants (Hymenoptera: Dasymutilla)”, Mendeley Data, V4, doi: 10.17632/dt7d7zjc9p.4
