# Supplementary material for: Evolutionary Drivers of Conspicuous Spots in Velvet Ants (Hymenoptera: Dasymutilla)
Source: Ecol Evol. 2025 Jan 30;15(2):e70896. doi: 10.1002/ece3.70896 (PMC11782072; doi:10.1002/ece3.70896)
Supplement: Supplementary file 1 — Appendix S1. [file ECE3-15-e70896-s001.pdf]

Evolutionary drivers of conspicuous spots in velvet ants (Hymenoptera: *Dasymutilla*)

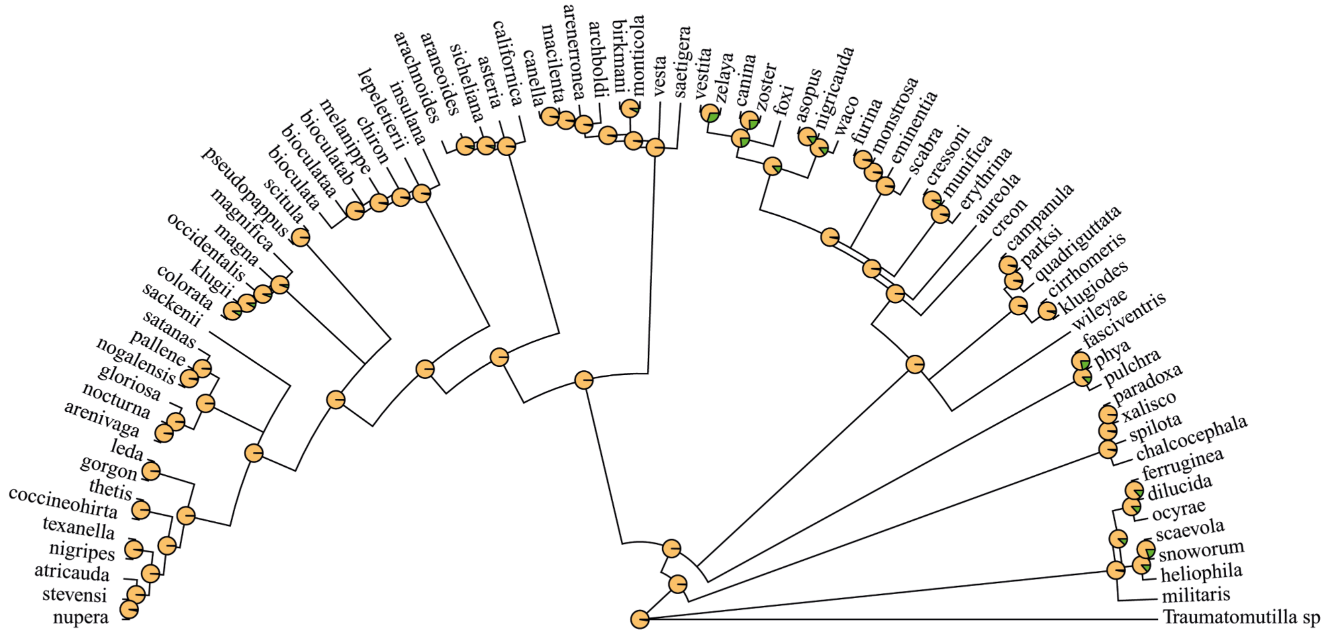

Figure S1: Ancestral state reconstruction for habitat occupancy. Generalist species is represented in green, and specialist species is represented in yellow.

Appendix Table S1. Matrix of probabilities for each state at the root nodes. (S) = spots, (F) = forest dwellers, (HG) = habitat generalists, (-) = absence of state, and (+) = presence of state. Bold values represent the most probable states.

**Models**	**States**			
	S. (-) F. (-)	*S. (-) F. (+)*	*S. (+) F. (-)*	*S. (+) F. (+)*
Forest habitat	0.19	0.03	0.23	**0.53**
	S. (-) HG. (-)	*S. (-) HG. (+)*	*S. (+) HG. (-)*	*S. (+) HG. (+)*
Habitat generalism	0.14	0.07	**0.49**	0.28

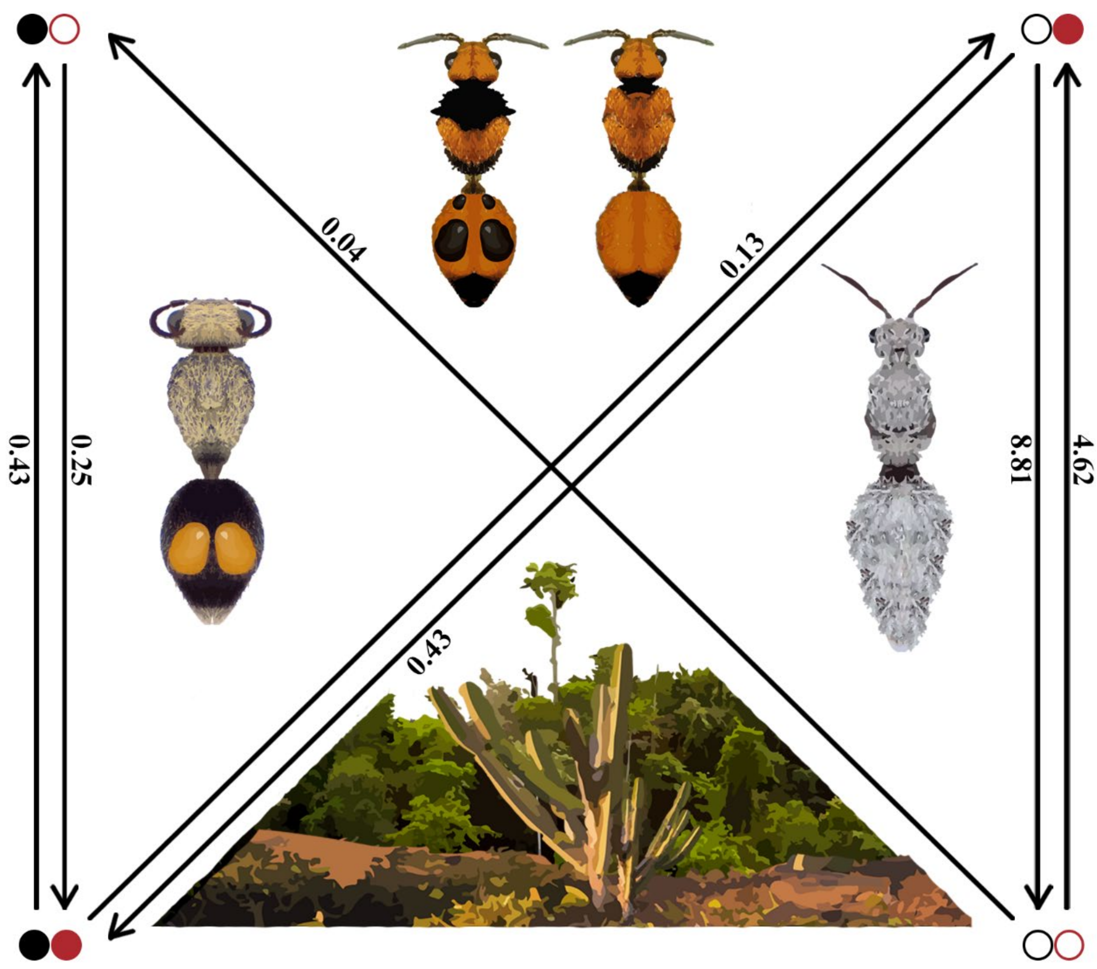

Figure S2. Evolutionary transition rates of conspicuous spots and generalist/specialist species. Black circles indicate presence of conspicuous spots, circles with black outline represent absence of conspicuous spots. Red circles denote presence in generalist species, and circles with red outline indicate absence in generalist species.

Table S2: values presented for various evolutionary models between conspicuous spots and open habitat. The columns denote a Markov model structure that may be consistent with independence or correlation. Bold indicates the model with the lowest AIC (Akaike Information Criterion). AICw = Akaike weight.

Markov model	Open areas	
	AIC	AICw
Independent	182.8	0.00
Correlated	**166.4**	0.88
Hidden Markov independent	170.5	0.11
Correlated hidden Markov	180.5	0.00

Table S3: Species used in phylogenetic logistic regression analyses, with the second column indicating the respective points counts for each species.

Species		Number per individuals
Dasymutilla	*arachnoides*	118
Dasymutilla	*araneoides*	56
Dasymutilla	*archboldi*	13
Dasymutilla	*arenerronea*	14
Dasymutilla	*arenivaga*	61
Dasymutilla	*asopus*	168
Dasymutilla	*asteria*	19
Dasymutilla	*atricauda*	14
Dasymutilla	*aureola*	3324
Dasymutilla	*birkmani*	19
Dasymutilla	*californica*	465
Dasymutilla	*campanula*	10
Dasymutilla	*canella*	11
Dasymutilla	*canina*	25
Dasymutilla	*chalcocephala*	12
Dasymutilla	*chiron*	441
Dasymutilla	*cirrhomeris*	10
Dasymutilla	*coccineohirta*	151
Dasymutilla	*colorata*	45
Dasymutilla	*creon*	91
Dasymutilla	*cressoni*	152
Dasymutilla	*dilucida*	9
Dasymutilla	*eminentia*	94
Dasymutilla	*erythrina*	552
Dasymutilla	*fasciventris*	13
Dasymutilla	*ferruginea*	10
Dasymutilla	*foxi*	228

Dasymutilla	*furina*	6
Dasymutilla	*gloriosa*	482
Dasymutilla	*gorgon*	189
Dasymutilla	*heliophila*	15
Dasymutilla	*klugii*	1577
Dasymutilla	*leda*	53
Dasymutilla	*lepeletierii*	661
Dasymutilla	*macilenta*	18
Dasymutilla	*magna*	136
Dasymutilla	*magnifica*	636
Dasymutilla	*melanippe*	22
Dasymutilla	*militaris*	9
Dasymutilla	*monstrosa*	24
Dasymutilla	*monticola*	18
Dasymutilla	*munifica*	58
Dasymutilla	*nigricauda*	6
Dasymutilla	*nigripes*	291
Dasymutilla	*nocturna*	42
Dasymutilla	*nogalensis*	30
Dasymutilla	*nupera*	32
Dasymutilla	*occidentalis*	8428
Dasymutilla	*ocyrae*	11
Dasymutilla	*pallene*	11
Dasymutilla	*paradoxa*	15
Dasymutilla	*parksi*	6
Dasymutilla	*pseudopappus*	78
Dasymutilla	*pulchra*	246
Dasymutilla	*quadriguttata*	1250
Dasymutilla	*sackenii*	1197
Dasymutilla	*saetigera*	22

Dasymutilla	*satanas*	86
Dasymutilla	*scabra*	6
Dasymutilla	*scaevola*	151
Dasymutilla	*scitula*	52
Dasymutilla	*sicheliana*	154
Dasymutilla	*snoworum*	48
Dasymutilla	*spilota*	12
Dasymutilla	*stevensi*	21
Dasymutilla	*texanella*	32
Dasymutilla	*thetis*	21
Traumatomutilla	*sp*	39
Dasymutilla	*vesta*	825
Dasymutilla	*vestita*	1369
Dasymutilla	*waco*	94
Dasymutilla	*wileyae*	12
Dasymutilla	*xalisco*	5
Dasymutilla	*zelaya*	183
Dasymutilla	*zoster*	10

Evolutionary drivers of conspicuous spots in velvet ants (Hymenoptera: *Dasymutilla*)

Methods

GenBank session codes

DQ408497, DQ408502, EF433451, EF433452, EU367350, EU627568 to EU627570, EU627572, HQ317244 to HQ317247, HQ317254, HQ317255, HQ317257, HQ317267, HQ317268, HQ317270, HQ317276, HQ317281, HQ317288, HQ317289, JQ945768 to JQ945771, JQ945773, JQ945776 to JQ945786, JQ945789 to JQ945792, JQ945794, JQ945797 to JQ945800, JQ945802 to JQ945805, JQ945807, JQ945808, JQ945811, JQ945834 to JQ945837, JQ945839, JQ945843 to JQ945851, JQ945858 to JQ945860, JQ945862, JQ945865 to JQ945869, JQ945871 to JQ945874, JQ945883 to JQ945885, JQ945887 to JQ945890, JQ945892 to JQ945896, JQ945899, JQ945909 to JQ945911, JX002743, JX002745 to JX002747, JX002775 to JX002777, JX002780 to JX002783, JX002785, JX002786, JX002789, JX002790, JX002792 to JX002794, JX002796, JX002797, JX002799, JX002800, JX002802 to JX002810, JX002812, JX002815 to JX002817, JX002820 to JX002853, JX002870, JX002887.

Logistic Regression Assumptions and Diagnostics

We follow the following assumptions and diagnostics. The outcome is a binary or dichotomous variable, such as yes vs. no, positive vs. negative, 1 vs. 0. The assessment of the linearity assumption was conducted by comparing the logit of the predicted probabilities with each predictor variable (Supplementary Figure S3). The smoothed scatter plots indicate that the logit is linearly associated with the predictor variables, thereby supporting the model's assumption of linearity. The influence analysis of the logistic regression model indicated no significant influential values. Cook's distance values were all below 1 (Supplementary Figure S4A), and standardized residuals remained under 3, suggesting the absence of outliers. The three most influential points exhibited standardized residuals of 2.28, 1.91, and -2.41, which are all within an acceptable range. Additionally, the variance inflation factor (VIF) values were low, indicating no significant multicollinearity issues (Tree cover = 1.026, Anuran diversity = 1.044, Avian diversity = 1.019) (Supplementary Figure S4B). Collectively, these findings suggest that the model is robust, and the estimates can be regarded as reliable.

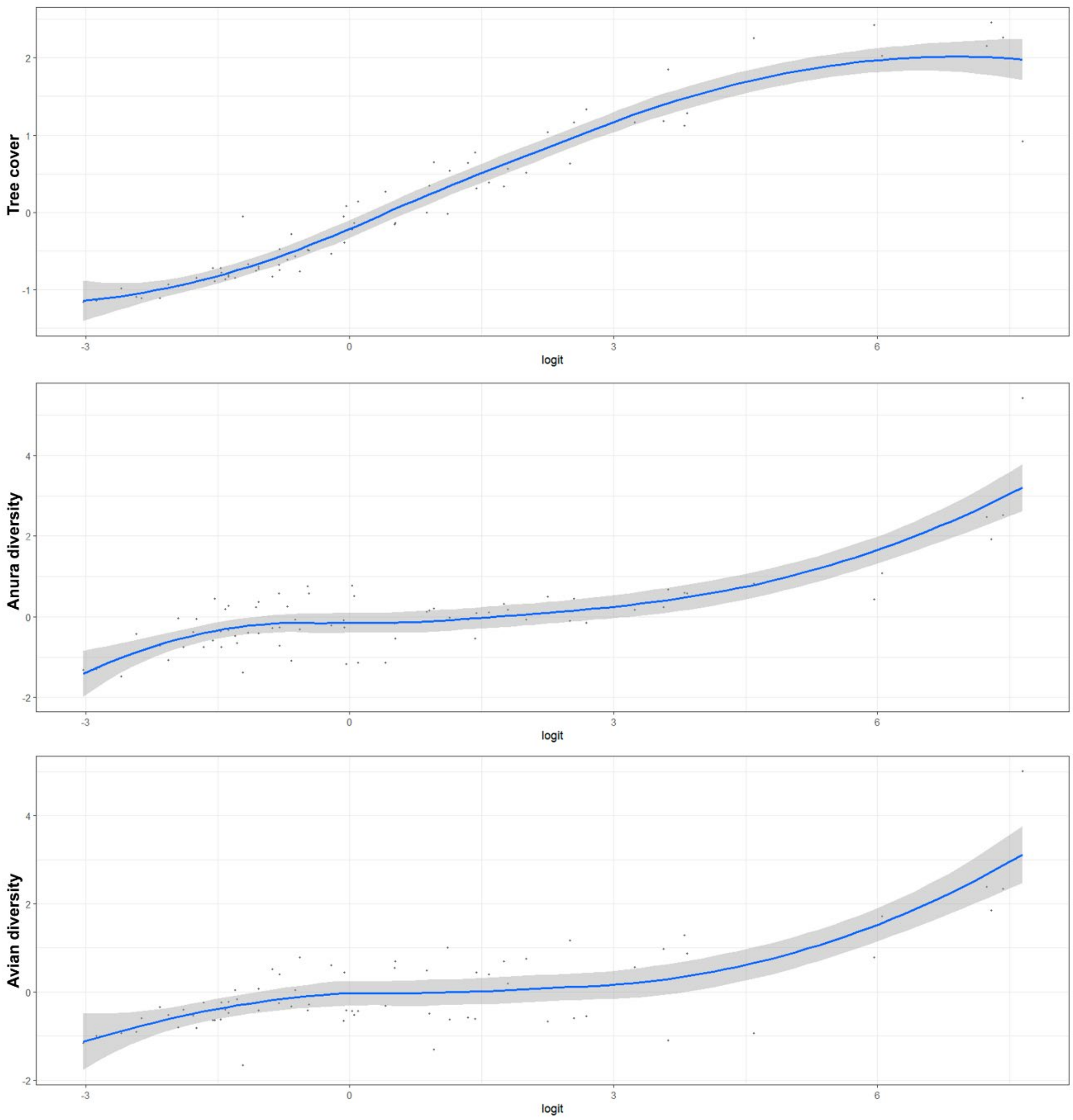

Figure S3. Smoothed scatter plots illustrating the relationship between the logit of predicted probabilities and each predictor variable (Tree cover, Anuran and Avian predator diversity). The plots demonstrate a linear association, supporting the model's assumption of linearity.

The influence analysis of the logistic regression model indicated no significant influential values. Cook's distance values were all below 1 (Supplementary Figure S4.A), and standardized residuals remained under 3, suggesting the absence of outliers. The three most influential points exhibited standardized residuals of 2.28, 1.91, and -2.41, which are all within an acceptable range. Additionally, the variance inflation factor (VIF) values were low, indicating no significant multicollinearity issues (Tree cover = 1.026, Anuran diversity = 1.044, Avian diversity = 1.019) (Supplementary Figure S4.B). Collectively,

these findings suggest that the model is robust, and the estimates can be regarded as reliable.

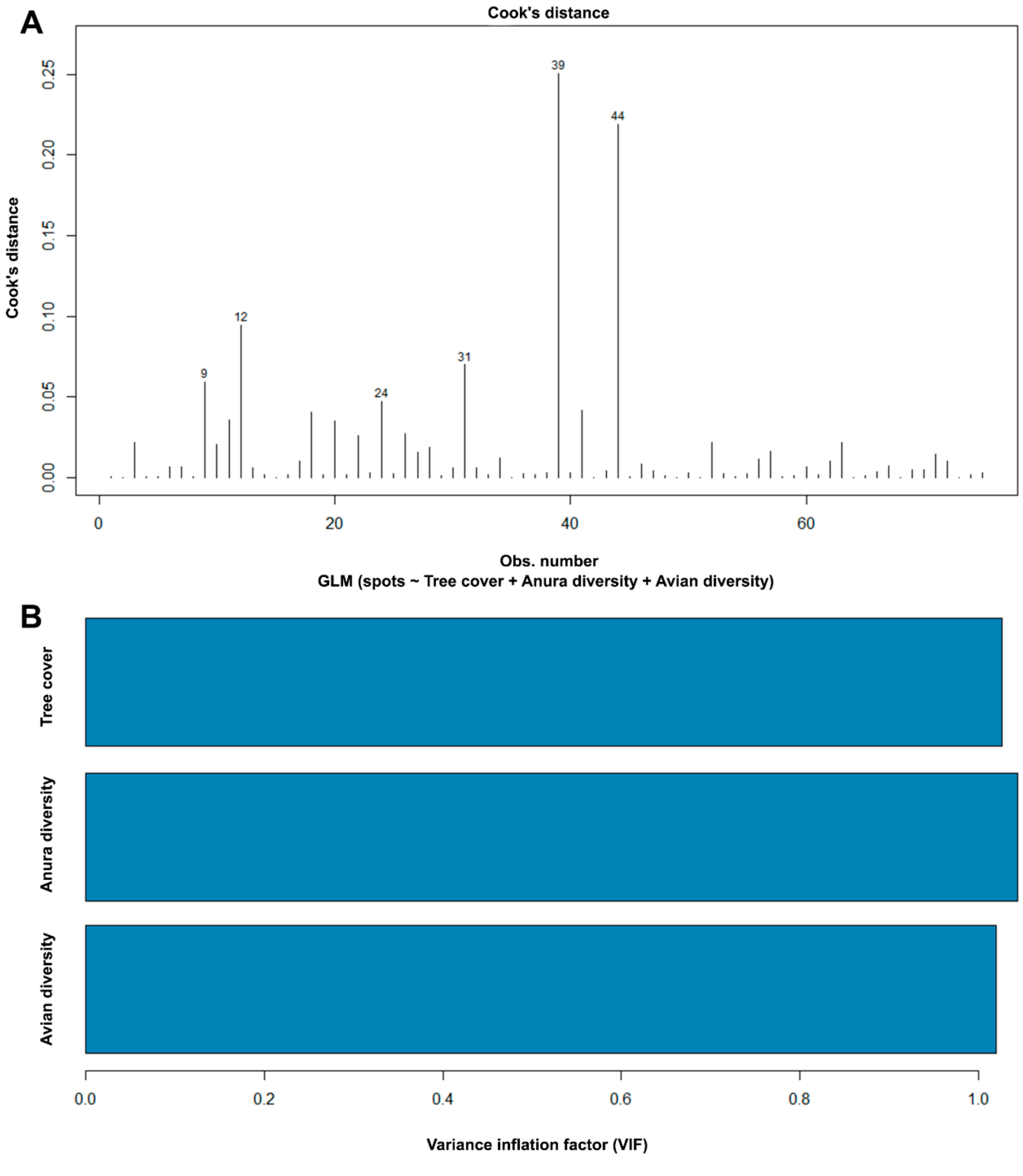

Figure S4. A. Cook's distance values for the logistic regression model, indicating no significant influential values, with all values below 1. B. Variance inflation factor (VIF) values for the predictor variables—Tree cover, Anuran and Avian predator diversity - showing no significant multicollinearity issues, with values of 1.026, 1.044, and 1.019, respectively.
